# Perioperative myocardial injury and infarction after noncardiac surgery: a review of pathophysiology, diagnosis, and management

**DOI:** 10.3389/fcvm.2024.1323425

**Published:** 2024-01-26

**Authors:** Besher Kashlan, Menhel Kinno, Mushabbar Syed

**Affiliations:** ^1^ Department of Internal Medicine, Loyola University Medical Center, Maywood, IL, United States; ^2^ Department of Cardiology, Loyola University Medical Center, Maywood, IL, United States

**Keywords:** myocardial injury, myocardial infarction, noncardiac surgery, troponin, risk assessment

## Abstract

Perioperative myocardial injury is a relatively common complication after noncardiac surgery associated with significant morbidity and mortality. It is frequently driven by physiologic factors such as hypotension, tachycardia, and anemia. Diagnosis of perioperative myocardial injury after noncardiac surgery is based on elevated cardiac troponin levels, greater than the 99th percentile of the assay's upper reference limit within 30 days of surgery. Perioperative myocardial injury is further classified into non-ischemic and ischemic based on the underlying pathophysiology. Ischemic injury, also called myocardial injury after non-cardiac surgery (MINS), is further classified into perioperative myocardial infarction or myocardial injury without infarction. Classifying perioperative myocardial injury further is particularly important for clinical management and prognosis. MINS—with or without infarction—is independently and strongly associated with short- and long-term mortality. Compared to nonoperative myocardial infarction, perioperative myocardial infarction carries an increased risk of adverse outcomes including all-cause mortality. Preventative measures include a thorough preoperative risk assessment, risk factor optimization, and avoidance of intraoperative mismatch of myocardial oxygen supply and demand. Surveillance of patients at higher risk of cardiovascular complications is warranted and can lead to early recognition, closer monitoring, and appropriate management. This review will provide a framework for understanding perioperative myocardial injury and highlight the contemporary literature addressing its diagnosis and management.

## Introduction

If perioperative mortality were judged independently, it would be the third leading cause of death worldwide, behind only ischemic heart disease and stroke (1). It is estimated that over 300 million patients worldwide undergo surgery yearly—an increase of over 100 million from 2 decades ago (2, 3). Among them, 3 percent are expected to have a major adverse cardiovascular and cerebrovascular event before discharge or within 30 days (4). Nearly one-third of these events are due to perioperative myocardial infarctions, corresponding to almost one myocardial infarction every 100 surgeries performed (4, 5). With an incidence over 10%, perioperative myocardial injury (with or without infarction) is a common contributor to short- and long-term morbidity and mortality of patients undergoing noncardiac surgery (5–9). Despite this elevated burden, there continue to be challenges and uncertainty around disease recognition and management, given its majority silent presentation (10).

## Definitions

**Figure 1:**
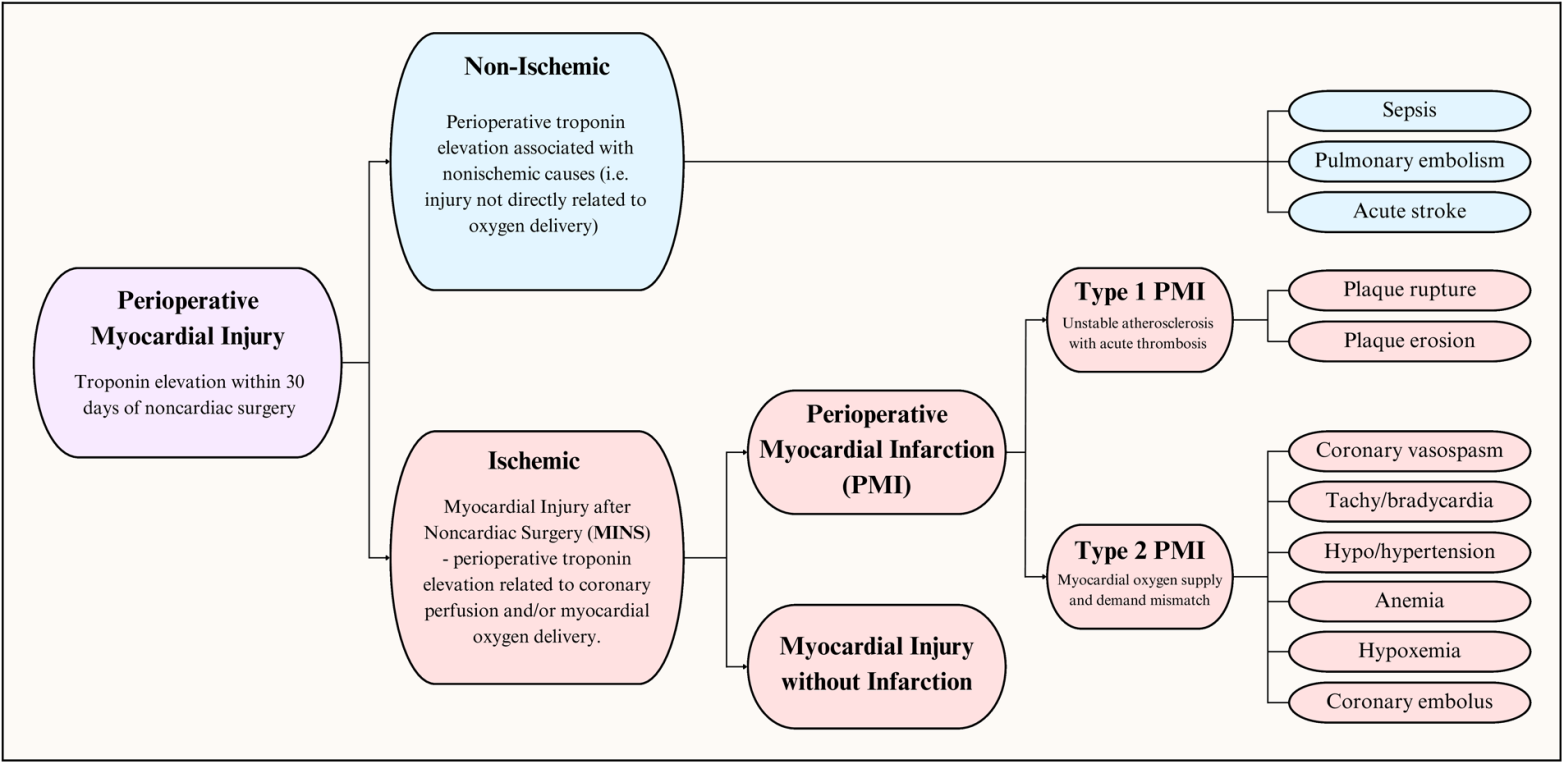
Perioperative myocardial injury: pathophysiological classification.

The syndrome of perioperative myocardial injury can be conceptualized as an insult resulting in cardiomyocyte injury, as evidenced by an increase in a cardiac injury biomarkers (Figure 1). Injury can be categorized as cardiac or extracardiac, otherwise referred to as ischemic and nonischemic (11). Based on the underlying pathophysiology, we prefer to use ischemic and non-ischemic injury rather than cardiac and extracardiac. Within the last decade, data from the Vascular Events In Noncardiac Surgery Patients Cohort Evaluation (VISION) study has been used to establish a new entity: Myocardial injury after noncardiac surgery (MINS). It is defined as myocardial cellular injury within 30 days of non-cardiac surgery deemed a consequence of an ischemic etiology (12). Non-ischemic causes of perioperative myocardial injury, such as sepsis and pulmonary embolism (PE), are excluded from the diagnosis of MINS. Simply put, MINS is a prognostically relevant, ischemic perioperative myocardial injury that encompasses both perioperative myocardial injury and infarction (12). Large cohort studies have demonstrated that perioperative myocardial infarction (PMI) constitutes less than half (20%–40%, dependent on cardiac troponin assay) of MINS cases (12, 13).

The 2018 Task Force for the Universal Definition of Myocardial Infarction on behalf of the European Society of Cardiology (ESC), the American College of Cardiology (ACC), the American Heart Association (AHA), and the World Heart Federation (WHF) define myocardial injury as evidence of elevated cardiac troponin values with at least one value above the 99th percentile upper reference limit. Myocardial injury can be chronic or acute in nature. Chronic myocardial injury can be used to describe a cardiac troponin that is stably elevated. In contrast, acute myocardial injury is characterized by an acute rise and fall of cardiac troponin level (14). Acute myocardial infarction (MI) is a type of acute myocardial injury wherein there is clinical evidence of myocardial ischemia resulting in myocardial cell death. Therefore, MI can be defined as a rise and fall in cardiac troponins with at least one of the following features (14):

• Ischemic symptoms (e.g., chest pain or pressure)
• New ischemic electrocardiographic (ECG) changes (e.g., ST-segment elevations/depressions, T-wave inversions)
• Development of pathologic Q-waves
• New regional wall motion abnormality or loss of previously viable myocardium on imaging (echocardiography, myocardial perfusion imaging)
• Identification of a coronary thrombus by angiography/autopsy

MI can be further categorized into five types reflecting mechanisms of infarction (Table 1) (14). PMI can be Type 1 or Type 2 based on the underlying mechanism and pathophysiology. Type 1 MI is the prototypical acute coronary syndrome due to rupture or erosion of a vulnerable plaque resulting in coronary thrombosis leading to partial or complete coronary vessel occlusion and regional myocardial ischemia. Type 2 MI, on the other hand, is not related to coronary athero-thrombosis and instead is a consequence of physiologic changes resulting in an imbalance between myocardial oxygen supply and demand (14, 15).

**Table 1:** Different types of myocardial infarction based on the fourth universal definition (14).

Type 1	MI secondary to spontaneous disruption or rupture of a vulnerable coronary plaque leading
Type 2	MI secondary to process that results in sustained imbalance between myocardial oxygen supply and demand
Type 3	Sudden cardiac death, which occurs before blood samples are obtained or resulted, accompanied by presumably new ST-segment elevation, new LBBB, or evidence of fresh coronary thrombus (via angiography and/or autopsy)
Type 4a	PCI-related MI
Type 4b	MI due to stent thrombosis any time after PCI
Type 4c	MI due to in-stent restenosis
Type 5	CABG-related MI

CABG, coronary artery bypass graft; LBBB, left bundle branch block; MI, myocardial infarction; PCI, percutaneous coronary intervention.

## Pathophysiology

The perioperative period boasts a myriad of unique physiologic changes that can predispose patients to increased myocardial demand and injury. This period is characterized by sympathetic activation, increased stress hormone production, and enhanced cytokine/acute phase reactant activity. The surge of catecholamines, combined with excess pituitary hormones such as adrenocorticotropic hormone (ACTH) and thyroid stimulating hormone (TSH), contributes to tachycardia, hypertension, and coronary vasoconstriction. Moreover, a hypercoagulable state results from systemic inflammation that alters platelet reactivity, procoagulant production, and fibrinolysis (15–17).

PMI is known to occur mainly via two main mechanisms. For one, the aforementioned physiologic changes can exact shear stress on a vulnerable or unstable plaque, promoting its disruption. This can result in acute coronary thrombus formation, leading to obstructed myocardial blood flow and subsequent infarction (Type 1 MI). In contrast to non-operative myocardial infarction, where most cases are Type 1 MI, a minority of peri-operative MI share this pathophysiology (18). Although a 2022 study demonstrated Type 1 MI to cause almost 60% of PMI (19), most other studies indicate that the majority are, in fact, Type 2 MI (20–22), wherein there is sustained mismatch between myocardial oxygen delivery and demand without underlying plaque rupture/erosion and thrombosis (6, 23). Under normal circumstances, the relationship between coronary blood flow and myocardial demand is almost perfectly linear (24). Oxygen delivery is reduced via factors such as coronary vasoconstriction, anemia, and hypoxemia, while demand increases in states of sustained tachycardia or hypotension (14). The effects of this mismatch extend beyond myocardial infarction and have been implicated in most MINS cases (11, 25). Although ischemic myocardial injury is classically categorized as Type 1 and Type 2 MI, Puelacher et al. suggest that further subtyping can guide disease prevention, treatment, and outcomes (21, 22). The proposed subtypes include Type 1 MI, tachyarrhythmia, acute heart failure, and Type 2 MI.

## Diagnosis

Cardiac biomarkers have long been used to identify myocardial injury (26). The detection of an abnormal elevation of such a biomarker, classically cardiac troponin, within 30 days of a noncardiac surgery can lead to the diagnosis of MINS (Table 2). Notably, the elevation must be attributed to ischemia (i.e., not due to nonischemic etiologies) for diagnosis of MINS.

**Table 2:** Myocardial injury after noncardiac surgery (MINS) diagnostic criteria and troponin assay prognostic thresholds (8, 12, 27).

Diagnostic Criteria	- Injury occurs within the first 30 days after noncardiac surgery. - At least one elevated troponin measurement >99th percentile of the URL, with rise/fall indicative of acute myocardial injury. - Myocardial injury attributed to ischemic etiology (i.e., Type 1 or Type 2 pattern of injury) in the absence of clear nonischemic etiology (i.e., sepsis, PE) - The presence of additional clinical symptoms or findings suggestive of ischemia is not required.
Prognostic Thresholds	- TnT ≥0.03 ng/ml - hsTnT ≥65 ng/L - hsTnT ≥20 and <60 ng/L with an absolute change ≥5 ng/L - hsTnT absolute change ≥14 ng/L

hsTnT, high-sensitivity troponin T; PE, pulmonary embolism; TnT, fourth generation troponin; URL, upper reference limit.

Diagnostic criteria for MINS were established based on the VISION study. Between 2007 and 2011, the study enrolled 15,000 patients for troponin T (TnT) surveillance within 30 days of noncardiac surgery (8). Using 30-day mortality data, researchers identified prognostically relevant threshold for MINS diagnosis (12). Over the years, high-sensitivity troponin assays have gained popularity due to their superior analytical performance compared to their earlier-generation counterparts (28). Between 2008 and 2013, VISION investigators recorded high sensitivity troponin T (hsTnT) levels in 21,000 patients and similarly identified a threshold for diagnosis. Preoperative hsTnT levels were also included for analysis to control for chronic troponin elevations (27).

Based on the assays used in VISION, prognostically relevant elevations in troponin levels were defined as TnT 0.03 ng/mL or greater, hsTnT 65 ng/L or greater, hsTnT ≥20 ng/L and <65 ng/L with an absolute change ≥5 ng/L, and absolute hsTnT change ≥14 ng/L. MINS is diagnosed within 30 days of surgery if an elevated troponin level (exceeding the used assay's 99th percentile of the upper reference limit) is identified and adjudicated to ischemic pathology (i.e., no discernible extracardiac etiologies) (12, 27).

As discussed, MINS is a diagnosis that also includes PMI. When a patient is found to have abnormally elevated troponin within 30 days of surgery and also displays at least one clinical feature of infarction—symptoms, ECG/imaging, or angiographic changes (as defined by the universal definition of MI)—the diagnosis of PMI can be made (14).

## Outcomes

### Short term outcomes

The prognostic relevance of MINS stems from its well-established impact on mortality. A 2019 meta-analysis of 195 studies published through November 2017 identified in-hospital mortality to be 8.1% [4.4–12.7%] among patients with MINS and 0.4% [0.2%–0.7%] among those without MINS (9). Risk of death within 30 days of surgery is 4–9 times higher if MINS is diagnosed (7, 9, 11, 12). The impact is not just limited to mortality, however. Patients with MINS are at higher risk of other complications, such as nonfatal cardiac arrest, congestive heart failure, and stroke. Clinical manifestation of disease may be important since VISION found 30-day mortality rates to be higher in MINS with an ischemic feature, symptoms, or ECG changes relative to without [13.5% (10.5–17.3) vs. 7.7% (5.7–10.2)] (12).

MINS that satisfies the universal definition of myocardial infarction (PMI) also significantly affects patient mortality and outcomes. PMI is linked to more extended average hospital stays (10 days vs. 3 days) and increased 30-day and 90-day readmission rates (19.1% vs. 6.5% and 36.2% vs. 17.2%, respectively) (19, 29). It is associated with higher rates of nonfatal cardiac arrest, congestive heart failure, and, unsurprisingly, mortality (10). Mortality is highest early in the disease course. Patients who experience PMI within the first 7 postoperative days have a higher risk of 30-day all-cause mortality than those who experience it within days 8–29 (30). Moreover, most patients who die from PMI do so within 48 h of diagnosis (10). This contributes to the higher observed in-hospital mortality rates, with a more than 12-fold increase, from 1.2% to 15.2%, relative to patients without PMI. More specifically, Type 1 MI is associated with a higher mortality rate when compared with Type 2 MI (17.4% vs. 12.1%) (19). PMI's positive association with death also extends to the 30-day mortality rates (10, 21). In contrast to MINS, the presence of ischemic symptoms does not portend a significantly worse prognosis when compared with silent presentations. The POISE trial found PMI without ischemic symptoms to be an independent predictor of 30-day mortality with an adjusted odds ratio (OR) of 4.00 [2.65–6.06] (10).

When comparing patients with MINS who fulfill the additional criteria for acute myocardial infarction to those with MINS who do not meet myocardial infarction criteria, comparable 30-day [8.7% (4.2–16.7) vs. 10.4% (6.7–15.7)] and 1-year mortality rates [29.1% (21.4–38.1) vs. 22.1 (17.6–27.5)] were found (11).

### Long-Term outcomes

Relatively few studies have addressed long-term outcomes of perioperative myocardial injury and infarction, but found that both MINS and PMI are associated with higher long-term mortality rates (9, 11). At 1 year, mortality is 20.6% [15.9–25.7] among patients with MINS and 5.1% [3.2–7.4] among patients without it. Although the relative risk (RR) of death with MINS trends down as time from surgery extends beyond 1 year (follow up ranging from 2 to 7 years), the mortality risk continues to be significant [RR, 2.4 (1.8–3.4)] (9).

It is clear that perioperative myocardial injury or infarction is associated with increased long-term complications relative to no infarction or injury. Still, the spectrum of etiologies also plays a significant role. Peulacher et al. compared major adverse cardiac event (MACE) and all-cause mortality rates after characterizing 7,754 patients with MINS into one of 4 categories: Type 1 injury, tachyarrhythmia, acute heart failure (AHF), and Type 2 injury (22). Median time from diagnosis to first MACE was significantly shorter for Type 1 injury (3 days) and AHF (5 days) when compared to Type 2 injury and tachyarrhythmia (13 and 14 days, respectively). Additionally, compared with other phenotypes, Type 2 myocardial injury/infarction is associated with significantly lower rates of MACE and all-cause mortality. This indicates that the etiology of myocardial injury both has implications on outcomes and alters the window of opportunity for potential intervention.

### Perioperative vs. nonoperative

As discussed above, the pathophysiology of PMI differs from nonoperative MI in that Type 2 MI makes up the majority of PMI cases (18). Korsgaard et al. examined long-term outcomes of PMI compared with nonoperative MI in a Danish cohort of over 5,068 patients with PMI and over 135,000 patients with nonoperative MI (30). MI was divided into ECG subtypes, ST-segment-elevation MI (STEMI) and non-ST-segment-elevation MI (NSTEMI), and PMI was identified in patients who had undergone noncardiac surgery on the date of admission or within 30 days before admission for MI. All-cause mortality was 64% in the PMI group and 35% in the nonoperative group. Adjusted relative risks (ARR) were 1.29 [1.23–1.36] at 30 days, 1.25 [1.21–1.30] at 1 year, and 1.13 [1.11–1.16] at 5 years. Trends demonstrated an early divergence in all-cause mortality rates within the first year, followed by a relative plateau. PMI was also associated with higher rates of venous thromboembolism and acute kidney injury (5-year ARR, 1.21 and 1.37, respectively). Mortality risk remained elevated in PMI patients when controlling for the urgency of surgery, the presence of a cancer diagnosis, or the presence of a STEMI. Reassuringly, over the course of the 16-year period that was analyzed, temporal trends reveal a decrease in risk estimates for all-cause mortality, cardiac mortality, and recurrent MI.

## Management considerations

### Risk assessment

Identifying patients who are at risk for developing myocardial injury can help guide shared clinical decision-making pre- and postoperatively. There are many considerations that contribute to a patient's likelihood of developing perioperative myocardial injury and infarction. Patient history factors include advanced age, male gender, cardiovascular diseases (such as coronary or peripheral artery disease, heart failure, and atrial fibrillation), and chronic medical conditions (such as diabetes mellitus, hypertension, chronic obstructive pulmonary disease, stroke, chronic kidney disease, and obstructive sleep apnea) (10, 12, 31, 32). Elevated preoperative creatinine, B-natriuretic peptide, and glucose concentrations are also associated with increased risk of MINS (10, 12, 33, 34). Moreover, the type of surgery itself and its urgent/emergent nature can similarly contribute to the development of MINS and PMI (10, 12, 22). The AHA stratifies surgery-specific risk for major cardiac events into high, intermediate, and low (35). High-risk surgeries include aortic, peripheral vascular, and emergent major surgery. Additionally, any procedure with a large, expected volume of blood loss or fluid shifts is considered high-risk, particularly in the elderly. Intermediate-risk surgeries include intraperitoneal, intrathoracic, orthopedic, head and neck, and prostate surgery. Low-risk surgeries include cataract, breast, superficial, and endoscopic procedures.

There are many prognostic models and risk indices used today that have well-validated performance in the prediction of MACE after noncardiac surgery. These include the Revised Cardiac Risk Index (RCRI), the American College of Surgeons National Surgical Quality Improvement Program Surgical Risk Score (ACS-NSQIP SRS), the Gupta Preoperative Risk Score (Myocardial Infarction/Cardiac Arrest Score or MICA), and the Goldman Risk Index, each with their unique advantages and pitfalls (36–38). Among them, only the RCRI—a widely recognized and simple-to-use model—has been well-correlated explicitly with MINS (22, 39). Although the NSQIP risk calculator has not been explicitly analyzed with respect to MINS, its utility cannot be understated since it enables surgery-specific risk assessment. Interestingly, higher scores on the STOP-Bang (Snoring, Tiredness, Observed Apnea, High Blood Pressure, Body Mass Index, Age, Neck Circumference, and Gender) risk score questionnaire for OSA were also independently associated with myocardial injury after noncardiac surgery (32). More specifically, high-risk scores (5 through 8) before major noncardiac surgery were associated with an age- and comorbidity-adjusted hazard ratio (HR) of 1.63 for the development of myocardial injury (*P = *0.03).

Currently, there is no universally accepted and well-validated risk stratification tool explicitly designed for the prediction of MINS. Using a machine learning analysis of 6,811 patients with normal preoperative troponin levels, Oh et al. developed two such models using 12 and 6 variables (40). Variables were selected based on clinical relevance and ease of access, and they include known risk factors such as nature of surgery (type of surgery and emergent vs. not emergent), patient age, and preoperative troponin levels. Accuracy, sensitivity, and specificity were 0.79, 0.29, and 0.93 in the 12-variable model and 0.79, 0.21, and 0.96 in the 6-variable model, respectively (40). Although promising, these models lack the sensitivity needed to be useful in clinical practice.

Current societal guidelines advise against the routine use of echocardiography in the preoperative assessment of asymptomatic and intermediate- to low-risk patients (41, 42). In a retrospective study, routine preoperative transthoracic echocardiography (TTE) was not a significant predictor of postoperative cardiovascular events (CVE) after noncardiac surgery (*P = *0.097) (43). However, abnormal TTE was an independent predictor for CVE occurrence (*P = *0.008).

More recently, left ventricular global longitudinal strain (LV-GLS), the echocardiographic measurement of subclinical systolic dysfunction, has demonstrated prognostic utility among patients with hypertrophic cardiomyopathy, heart failure, and valvular disease (44–47). In the SOLOMON study, Kim et al. prospectively observed the prognostic value of LV-GLS in 871 patients undergoing noncardiac surgery between 2020 and 2022, the majority of whom were low- to intermediate-risk (48). Groups were divided into “impaired” LV-GLS (<16.6%) and “not impaired” LV-GLS (≥16.6%). Although RCRI was not different between the two groups, impaired LV-GLS was associated with a higher 30-day incidence of the composite outcome of all-cause death, acute coronary syndrome (ACS), and MINS, as well as the composite outcome of all-cause death and ACS. When comparing patients with MINS to those without, LV-GLS values varied significantly (15.9% vs. 17.4%; *P = *0.003), and thus LV-GLS displays great promise as a predictive tool for MINS [OR, 3.54 (1.70–7.36)].

It remains unclear which patients would benefit from pre-operative TTE. The PREOP-ECHO study is an ongoing trial evaluating the effects of TTE on perioperative management and postoperative outcomes in intermediate- and high-risk patients (49). Researchers will randomize 2,330 intermediate-risk patients to receive preoperative echocardiography or not. High-risk patients will instead be enrolled in a prospective cohort study, in which the use of TTE will be at the discretion of the physicians responsible for their care. Of note, LV-GLS will be measured in every patient in the TTE arm. The primary outcome will be 30-day composite and all-cause mortality, aborted sudden cardiac arrest, type 1 MI, unstable angina, stress cardiomyopathy, lethal arrhythmias, and acute HF. MINS in the absence of MI will not be observed. Researchers hypothesize that TTE will reduce postoperative cardiovascular events in both intermediate- and high-risk populations. The results of this study have the potential to impact future guidelines around the more widespread use of TTE in the preoperative period.

The American College of Cardiology and The American Heart Association have published guidelines recommending a stepwise approach to preoperative cardiac assessment for coronary artery disease prior to noncardiac surgery, including appropriate testing (41, 42). In summary, asymptomatic patients planned for low-risk surgery generally do not require routine testing. For patients with pre-existing cardiac disease undergoing intermediate- or high-risk surgery, a resting 12 lead ECG is recommended. It is reasonable to pursue echocardiography in patients with clinical presentations suggestive of unstable coronary artery disease or heart failure. Stress testing can be performed before high-risk noncardiac surgery for patients with elevated risk and poor functional capacity, provided the results will change management. In the absence of symptoms or abnormal preoperative stress testing, as described, routine invasive angiography is not advised prior to noncardiac surgery, regardless of risk.

### Troponin surveillance for MINS

Since patients in the postoperative period are often on some degree of pain control, it is common for perioperative myocardial injury to be unaccompanied by symptoms. A large majority of patients (82%–93%) diagnosed with MINS and over 60% of patients diagnosed with PMI are completely asymptomatic (10–12, 27). Despite being clinically silent, MINS and PMI carry increased mortality risk, regardless of whether or not symptoms are present (10, 12). This makes recognition and diagnosis difficult without routinely measuring cardiac troponin levels. Furthermore, myocardial injury occurs at the highest rate within the first 72 h from surgery (8, 10), making it an ideal window to screen patients. Consequently, as highlighted in a 2021 statement from the AHA, there is broad consensus that routine postoperative surveillance of troponin levels is recommended for high-risk patients (50). The ESC gives a class IIb recommendation favoring postoperative measurement of high-sensitivity troponin levels in high-risk patients (METs ≤4 or RCRI >2) undergoing nonvascular surgery, although the frequency and duration of measurements are not defined (51). Moreover, the Canadian Cardiovascular Society recommends daily troponin measurements for up to 72 h after noncardiac surgery for high-risk patients, defined as >5% risk for cardiovascular death or nonfatal MI at 30 days after surgery (roughly RCRI ≥2) (52). Its usefulness in lower-risk patients, however, continues to be unclear.

Daily troponin measurements would contribute additional costs to medical care, although the cost per health gain may be appealing. One analysis of over 6,000 Canadian patients enrolled in the VISION study suggests the incremental cost to avoid missing a MINS event through TnT screening to be less than 1,650 CAD (2015 Canadian Dollar) (53). Since the VISION study did not have a standard-of-care alternative nor did it collect data on resource use, researchers made assumptions that underestimated the cost of standard care, subsequently overestimating the relative cost associated with TnT screening. Also, using incidence and mortality data from the VISION study, Torborg et al. conducted a pharmacoeconomic analysis of patients undergoing noncardiac surgery in South Africa (54). They assumed a 25% relative risk reduction for cardiovascular mortality and fatal myocardial infarction after treatment with Aspirin and statins; however, they did not consider MINS that did not fulfill the criteria for myocardial infarction. When comparing the expected cost of care for a patient surveilled and treated postoperatively to the expected cost of care for a patient who would not receive TnT surveillance, they found an incremental increase in cost per patient of 320.86 ZAR (2014 South African Rand), or 29.6 USD [2014 USD, based on average exchange rate (55) in 2014]. The total incremental cost to avoid perioperative myocardial infarction after noncardiac surgery was 32,409.80 ZAR (or 2,991.4 USD), which is less than the average per capita contribution to the South African GDP. Thus, troponin surveillance, allied to aspirin and statin therapy, was considered to be potentially cost-effective (54). Our literature search revealed no such studies in the US population.

The cost of screening, among other factors, has led to continued debate around routine postoperative troponin measurements in asymptomatic patients (56). For one, the context of preoperative troponin levels must be considered. This is particularly true in patients with pre-existing renal dysfunction, diabetes, hypertension, and left ventricular hypertrophy since they often have elevated TnT concentrations at baseline (57). Based on the current literature, screening troponin measurements should be considered in high-risk patients (RCRI ≥2, 10% 30-day risk of death, MI, or cardiac arrest) after noncardiac surgery, preferably getting a baseline value prior to surgery for comparison.

### Prevention strategies

The multivariable etiology of MINS poses a management challenge since treatment strategies will differ based on the underlying etiology and other clinical factors. From a prevention standpoint, avoiding adverse vital sign changes is paramount since intraoperative tachycardia, hypertension, and hypotension are associated with an increased risk of MINS (25, 58). Perioperative hypotension—minimum of 13 min with MAP <65 mmHg—and tachycardia—a mere increase in heart rate of 10 bpm from baseline—are independent predictors of perioperative myocardial injury and infarction with an odds ratio (OR) of 1.34 [1.06–1.68] and 1.29 [1.13–1.50], respectively (10, 58). This is consistent with a secondary analysis of VISION, which demonstrated an association between HR >100 bpm as well as SBP <100 mmHg and MINS (OR, 1.27 and 1.21, respectively) (25).

Perioperative hemoglobin concentrations can also contribute to cardiac myocyte injury since MINS is predominantly a disease of mismatched myocardial oxygen supply and demand. In a single-center retrospective study of 35,170 patients who underwent noncardiac surgery, preoperative anemia (hemoglobin <13 g/dl) was associated with a more-than-twofold increase in MINS incidence (23.5% vs. 11.5%; *P *< 0.001) (59). Turan et al. retrospectively analyzed 4,480 patients who had hemoglobin concentration obtained within the first 3 postoperative days after noncardiac surgery (60). Lowest postoperative hemoglobin concentration was inversely related to MINS incidence, and the hazard ratio for having MINS was 1.29 [1.16–1.42] for every 1 g/dl drop from preoperative to postoperative hemoglobin.

It is undetermined whether cardiovascular medications should be initiated preoperatively for MINS prevention (Table 3). The largest trial addressing the use of perioperative beta-blockers is the Perioperative Ischemic Evaluation (POISE) trial, which randomized 8,351 patients to receive either extended-release metoprolol or placebo. Intervention arm patients received 100 mg metoprolol 2–4 h preoperatively, a second dose up to 6 h postoperatively, and maintained on 200 mg daily thereafter for 30 days. Long-acting metoprolol use was associated with a reduction in cardiovascular mortality [HR, 0.84 (0.70–0.99)]; however, its use also increased the risk of stroke [HR, 2.17 (1.26–3.74)] as well as clinically significant hypotension and bradycardia [HR, 1.55 (1.38–1.74) and HR, 2.74 (2.19–3.43), respectively] (61). Concerns have been raised about the POISE trial design mainly due to the higher-than-normal initial dose used that likely led to adverse effects (62). More recently, a retrospective cohort study analyzed over 200,000 patients who underwent major abdominal surgery (high-risk, noncardiac surgery as defined by RCRI) and categorized them based on beta-blocker exposure: naïve (no beta-blocker therapy), preoperative (beta-blocker therapy initiated <60 days before surgery), and chronic (beta-blocker therapy started >60 days before surgery) (63). When compared to beta-blocker naïve patients, stroke risk was similar for patients who initiated beta-blocker therapy within 60 days before surgery [OR, 0.90 (0.31–2.04)] and patients on chronic beta-blocker therapy [OR, 0.86 (0.65–1.15)]. All-cause mortality and myocardial infarction were lower in patients on chronic beta-blocker therapy relative to beta-blocker naïve patients.

**Table 3:** Societal guidelines for perioperative pharmacologic therapy for noncardiac surgery (40, 41).

	ACC/AHA 2014	ESC 2022
Beta Blockers	- Continue in patients chronically receiving therapy. (Class I) - Can consider initiation in high-risk patients (RCRI ≥3) and those with intermediate- to high-risk ischemia on preoperative testing (Class IIb)	- Continue in patients chronically receiving therapy. (Class I) - Can consider initiation in advance of high-risk NCS in patients with known CAD or ≥2 clinical risk factors. (Class IIb)
Statin	- Continue in patients chronically receiving therapy. (Class I) - Can consider initiation in advance of high-risk NCS in patients with indication for statin. (Class IIb)	- Continue in patients chronically receiving therapy. (Class I) - Can consider initiation in patients with indication for statin. (Class IIa)
Aspirin	- Continue in patients with history of PCI, if possible. (Class I) - Continuation may be reasonable in patients on therapy for primary prevention, provided bleeding risk allows for it. (Class IIb)	- Continue in patients with history of PCI, if the bleeding risk allows for it. (Class I) - To reduce bleeding risk, consider interruption ≥3 days prior to NCS in patients without history of PCI. (Class IIb)
ACE/ARB	- Continuation is reasonable. (Class IIa)	- To prevent peri-operative hypotension, consider interruption the day of NCS in patients without heart failure. (Class IIa) - Consider continuation in patients with stable heart failure. (Class IIb)

ACE, angiotensin-converting enzyme inhibitor; ARB, angiotensin receptor blocker; CAD, coronary artery disease; NCS, noncardiac surgery; PCI, percutaneous coronary intervention; RCRI, revised cardiac risk index.

Despite the well-established benefit of statin use for both treatment and primary prevention of atherosclerotic cardiovascular disease (ASCVD) (64–66), data about initiation in the immediate preoperative period for MINS or PMI prevention is limited. The Lowering the Risk of Operative Complications Using Atorvastatin Loading Dose (LOAD) trial is a multicenter trial that randomized 648 statin-naïve Brazilian patients to either receive atorvastatin or placebo (67). An 80 mg loading dose of atorvastatin was administered within 18 h before surgery, followed by 40 mg daily for the next 7 days. At 30 days after randomization, risks were comparable between the two groups for all-cause mortality [HR, 1.14 (0.53–2.47)], myocardial infarction [HR, 0.76 (0.35–1.68)], and MINS [HR, 0.79 (0.53–1.19)]. Although not statistically significant, hazard ratios for PMI and MINS appeared to trend toward a reduction in risk. Since the study was relatively underpowered, the potential remains for a larger, international randomized control trial (RCT) to demonstrate statistically significant benefit.

Antiplatelet therapy with aspirin has proven beneficial for secondary prevention of ASCVD. However, its use for primary prevention has recently been questioned, especially in older adults, and remains controversial (64–66, 68, 69). Moreover, as previously discussed, bleeding risk is inherently greater in the perioperative period. The POISE-2 trial randomized 10,010 patients preparing to undergo noncardiac surgery to receive a combination of Aspirin vs. placebo and clonidine vs. placebo (70, 71). Patients were assigned to either receive placebo or 200 mg Aspirin just before surgery, followed by continued therapy for 30 days (30 days of placebo vs. 30 days of 100 mg Aspirin). Using a 2 × 2 factorial design, patients were also randomized to receive 0.2 mg oral clonidine or placebo 2–4 h before surgery, followed by a clonidine or placebo patch to remain on until 72 h after surgery (3 days of clonidine vs. 3 days of placebo). Primary outcome was composite death or nonfatal MI. Neither aspirin nor clonidine administration significantly affected primary outcome development. However, aspirin use was associated with greater major bleeding risk [HR, 1.23 (1.01–1.49)] (70), and clonidine use was associated with higher rates of nonfatal cardiac arrest [HR, 3.20 (1.17–8.73)], clinically important hypotension [HR, 1.32 (1.24–1.40)], and clinically important bradycardia [HR, 1.49 (1.32–1.69)] (71). Neither antiplatelet nor alpha-blocker therapy can reasonably be used for PMI prevention.

Angiotensin-converting enzyme inhibitors (ACE) and angiotensin II receptor blockers (ARB) can theoretically contribute to intraoperative hypotension. Studies have reported their use to be associated with an increased risk of MINS (9). Moreover, secondary analysis of VISION suggested that withholding ACE/ARB therapy prior to surgery can reduce the risk of MINS [RR, 0.84 (0.70–0.998)] (72). The STOP-or-NOT trial (NCT03374449) is an ongoing open-label RCT in over 30 French centers, evaluating the effects of stopping ACE/ARB therapy 48 h before noncardiac surgery when compared to the continuation of therapy (73). Primary endpoint is a composite of all-cause mortality and major postoperative complications (including AMI, but not all MINS). The results of this trial should be able to address the issue of perioperative management of ACE/Arb before major noncardiac surgery.

An important ongoing RCT for perioperative cardioprotection strategies is POISE-3 (NCT03505723): a multicenter, international trial that plans to enroll 10,000 adults with cardiovascular disease undergoing noncardiac surgery (74). Investigators will be exploring tranexamic acid vs. placebo (normal saline) for the prevention of major bleeding events within 30 days. Primary safety outcome is a composite outcome of MINS, non-hemorrhagic stroke, peripheral artery thrombosis, and symptomatic proximal venous thromboembolism. Secondary outcomes include MINS and MINS not fulfilling the universal definition of MI. Using a 2 × 2 factorial design (similar to POISE-2) for patients chronically taking antihypertensive medications, researchers will simultaneously compare perioperative hypotension-avoidance strategies (hold home antihypertensives pre- and postoperatively in the absence of significant hypertension; target intraoperative MAP >80 mmHg) to hypertension-avoidance strategies (give all home antihypertensives pre and postoperative; target intraoperative MAP >60 mmHg). Primary outcome for the BP management arm of the trial is the composite outcome of vascular death and non-fatal MINS, stroke, and cardiac arrest at 30 days. Secondary outcomes also include MINS and MINS not fulfilling the universal definition of MI. Results of the latter half of the trial will carry significant potential to further guide preoperative management strategies with respect to myocardial injury.

### Treatment strategies

Management of patients after diagnosis of MINS continues to elude consensus and is an area of ongoing investigation. Employment of a multidisciplinary model, in which surgeons and internists/hospitalists collaborate on patient care, can improve perioperative outcomes (75, 76). As an example, admission to orthogeriatric service—in which comprehensive geriatric/internist assessment was readily available and ECGs were performed routinely by nursing—after hip surgery was associated with significant reductions in in-hospital (*P *< 0.0001), 30-day (*P *= 0.003), 90-day (*P *= 0.002), and 1-year mortality (*P *= 0.006) (77). As an extension of co-management, early referral to inpatient cardiology services can also reduce the expected risk of death (78). Experts emphasize the importance of a multidisciplinary approach focused on care of multi-morbidity, hemodynamic monitoring, anticoagulation strategies, and the prevention of extracardiac factors such as infection and bleeding (79).

Early introduction or increase of cardiovascular pharmacotherapy after MINS can reduce rates of adverse cardiac events (80), but specific directions remain uncertain. Some studies have investigated the role of aspirin and statins, many of which demonstrated that their use could reduce mortality in both MINS and PMI (10, 80–82). Although lacking targeted randomized trials for these medications, evidence is limited to retrospective and post-hoc analyses. In the 415 patients who suffered PMI in POISE, in-hospital use of aspirin was associated with a reduction in mortality risk within 30 days [OR, 0.54 (0.29–0.99)] (10). Statin use in this cohort was also associated with a similar mortality risk reduction [OR, 0.26 (0.13–0.54)]. This finding is consistent with other studies. One such study of 5,109 patients with MINS revealed that—when adjusted for age, gender, comorbidities, surgical risk, and intraoperative interventions—statin use was associated with a reduction in 1-year mortality from 13.3% to 6.1% [HR, 0.61 (0.50–0.74)] (83). Though no RCT currently exists that directly evaluates their use in the setting of MINS, it seems that aspirin and statins may benefit patients with MINS, and thus it is strongly recommended by the Canadian Cardiovascular Society (52). The anti-inflammatory effects of glucocorticoids have also been hypothesized to reduce post-surgical cardiac stress. However, a preliminary prospective study of 290 patients found no effect of dexamethasone on postoperative troponin concentrations (84).

Antithrombotic therapy has proven benefit and is a recommended treatment for acute coronary syndromes not requiring invasive intervention (65, 85), and it appears to have similar effects in perioperative myocardial injury. The Dabigatran in Patients with Myocardial Injury After Non-Cardiac Surgery (MANAGE) trial randomized 1,754 patients with MINS to receive either dabigatran 110 mg twice daily or placebo with a primary efficacy outcome of composite risk of vascular mortality and non-fatal MI, non-hemorrhagic stroke, peripheral arterial thrombosis, amputation, and symptomatic VTE (86). As discussed above, 91% of observed MINS events occurred silently without clinical signs of ischemia. Dabigatran use was associated with a 25% relative reduction of major vascular complication risk with a hazard ratio of 0.72 [0.55–0.93]. Among secondary efficacy outcomes, risk reduction remained significant only for non-hemorrhagic stroke [HR, 0.20 (0.04–0.90)]. This benefit was seen without an increased risk of life-threatening, major, or critical organ bleeding (86). Moreover, dabigatran use was cost-neutral relative to placebo (87). Acknowledging limitations, including a high rate of treatment discontinuation and a lowering of the target enrollment, MANAGE provides a potential direction for MINS therapy. Further studies are needed to define optimal treatment, but early moderate-intensity anticoagulation with dabigatran may provide benefit in MINS.

The role of invasive management of MINS is unclear, however, experts suggest that it may be beneficial for patients with high-risk features (e.g., PMI, marked troponin elevations, persistent electrocardiographic or ischemic changes on imaging) (50). In a propensity-matched cohort of 34,650 patients with PMI, an invasive approach was associated with lower in-hospital mortality than a conservative approach (8.9% vs. 18.1%; *P *< 0.001) (5). However, it was also associated with increased rates of postoperative hemorrhage. Despite the potential benefits, in clinical practice, invasive management in PMI remains relatively low. In a group of almost 85,000 patients diagnosed with PMI between 2005 and 2013, only 21% underwent angiography. Among them, 37% underwent some form of revascularization, most commonly PCI (5). More recently, a Danish cohort found that 38.5% of patients with PMI underwent coronary angiography vs. 72% of patients with nonoperative MI (30). Perhaps the pattern of adopting a conservative management approach can be explained by the lower proportion of STEMI among patients with PMI and a higher proportion of non-specific myocardial injury. Compared to nonoperative myocardial infarctions, PMIs are comprised of half as many STEMIs (12% vs. 25%) (30). Though a case can be made for the adoption of a more aggressive and invasive management approach in patients with PMI with high-risk features, further evaluation of benefits is needed.

## Discussion

Although the body of literature surrounding perioperative myocardial injury is vast, the management recommendations are limited, as discussed. Most of the patients included in the above-mentioned studies are considered intermediate- to high-risk, and thus, our recommendation applies mainly to that population.

Preoperative cardiovascular risk assessment should be performed during surgical planning, and cardiac conditions should be optimized before surgery if possible. Important risk factors to consider include advanced age, male sex, functional capacity (Duke Activity Status Index ≤34), atherosclerotic disease (coronary or peripheral artery disease) and its associated risk factors (hypertension, diabetes), as well as other comorbidities (chronic kidney disease, obstructive sleep apnea). Predictive indices and scoring systems that have been independently associated with MINS include RCRI and STOP-Bang. Since RCRI is the most widely used tool for rapid preoperative risk assessment overall, we advocate for its use in the preoperative setting for patients ≥45 years of age or <45 years with multiple comorbidities. In the absence of large-scale validation studies, we cannot recommend the use of the previously discussed MINS-specific predictor designed by Oh et al. In patients identified as high-risk, expert consultation should be strongly considered.

Although preventative measures will differ for each patient, maintaining homeostasis while avoiding disruptions in myocardial oxygen delivery (e.g., persistent hypotension, hypertension, tachycardia, hypoxia, and anemia) is paramount for prevention of MINS. Results of preoperative risk assessment, comorbid conditions, and chronic medications are used to inform an individualized prevention strategy with consideration of expert consultation wherever appropriate. The current body of evidence does not support using anti-ischemic drugs to mitigate MINS; however, patients being chronically treated with beta-blockers (>60 days before surgery) will likely benefit from therapy continuation. Due to the increased risk of bleeding, we advise avoidance of aspirin initiation for the sole purpose of primary MINS prevention. The results of the ongoing POISE-3 trial will provide insight into hypotension-avoidance vs. hypertension-avoidance strategies in noncardiac surgery, the utility of TXA for bleeding prevention in these patients, and the effects these interventions have on MINS development.

After surgery, intermediate to high-risk patients should be managed jointly by surgeons and internists, with the help of a cardiology consultant when appropriate, on a co-management service in order to reduce adverse outcomes. In patients with scores ≥2 on the RCRI or those with reduced exercise capacity METS ≤4, we advocate for postoperative screening for MINS with serial troponins, as recommended by European and Canadian societies. Daily troponin concentrations should be measured for the first 72 h after surgery in these patients. Selection of troponin assays and prognostic threshold will differ based on the institution's available assay. Since high-sensitivity troponin assays have superior analytical performance, they should be the preferred assays. Nonetheless, if any postoperative troponin concentration is found to be above the 99th percentile of the upper reference limit for the respective assay, values should then be trended to peak concentrations. Nonischemic causes of troponin elevations (e.g., sepsis, pulmonary embolism) should be carefully ruled out to facilitate the diagnosis of MINS. Prompt identification of the cause of myocardial injury is critical since etiology has implications for management.

The optimal pharmacologic strategy for MINS not satisfying the universal definition of myocardial infarction is uncertain and largely based on observational studies. Nonetheless, intensification of the cardiovascular regimen (antiplatelet drugs depending on bleeding risk, statin, beta blocker, or ACE inhibitor) is recommended. Specific treatment with aspirin and statin therapy is thought to provide benefit, especially since these medications have proven beneficial for the prevention of adverse outcomes in patients with known cardiovascular diseases (64, 88). Although MANAGE showed promising results favoring moderate-intensity anticoagulation with dabigatran, it has not yet been approved for use in MINS treatment. Invasive strategies are usually reserved for patients who satisfy criteria for perioperative MI but can be considered in MINS with high-risk features (heart failure, reduced ejection fraction, ventricular arrhythmia, prior revascularization).

It is important to distinguish the subset of patients with MINS who satisfy the universal definition of myocardial infarction (PMI) since current guidelines for acute coronary syndrome can be better applied to these patients. Provided benefits outweigh the risks, patients should be initiated on appropriate guideline-directed medication therapy for spontaneous MI as per societal recommendations. Current guidelines dictate that all patients with spontaneous myocardial infarction will likely benefit from the initiation of beta-blocker, antiplatelet, statin, and ACE inhibitor/ARB therapy (64, 65). Expert opinions suggest that early angiography and invasive interventions should certainly be utilized for patients with STEMI (89), but can also be considered in other high-risk patients with PMI.

Patients with MINS need close follow-up after discharge for optimization of medical therapy and further risk stratification based on their clinical risk and type of MINS (myocardial injury vs. MI).

## Conclusion

Perioperative myocardial injury (MINS and PMI) is a common and prognostically relevant syndrome that affects more than one of every ten patients undergoing noncardiac surgery. It is associated with a significantly increased risk of mortality and cardiovascular complications. PMI compared to nonoperative MI carries an increased mortality risk. However, PMI has a relatively silent clinical presentation in the setting of anesthesia/pain management, and thus a high index of suspicion is needed. Since injury most commonly occurs in the first 72 h after surgery, high-risk patients may benefit from routine screening with daily troponin measurements during this time. Atherosclerotic risk factor optimization and avoidance of perioperative factors that can alter myocardial oxygen delivery (e.g., hypotension, hypertension, tachycardia, bradycardia, anemia, hypoxemia) are paramount for the prevention of MINS. Treatment remains uncertain and should be individualized based on the etiology and the patient's clinical status/risk.
